# Evidence of Skyrmion-Tube Mediated Magnetization Reversal in Modulated Nanowires

**DOI:** 10.3390/ma14195671

**Published:** 2021-09-29

**Authors:** E. Berganza, J. Marqués-Marchán, C. Bran, M. Vazquez, A. Asenjo, M. Jaafar

**Affiliations:** 1 Institute of Nanotechnology (INT), Karlsruhe Institute of Technology (KIT), Hermann-von-Helmholtz-Platz 1, 76344 Eggenstein-Leopoldshafen, Germany; 2 Instituto de Ciencia de Materiales de Madrid (ICMM), Consejo Superior de Investigaciones Cientificas (CSIC), Campus de Cantoblanco, C. Sor Juana Inés de la Cruz 3, 28049 Madrid, Spain; jorge.marques@csic.es (J.M.-M.); cristina.bran@icmm.csic.es (C.B.); mvazquez@icmm.csic.es (M.V.); aasenjo@icmm.csic.es (A.A.); 3 Departamento de Física de la Materia Condensada and Condensed Matter Physics Center (IFIMAC), Universidad Autónoma de Madrid, Avda. Francisco Tomás y Valiente 7, 28049 Madrid, Spain; miriam.jaafar@uam.es; 4 Instituto Nicolás Cabrera, Universidad Autónoma de Madrid, Avda. Francisco Tomás y Valiente 7, 28049 Madrid, Spain

**Keywords:** magnetic nanowires, skyrmion tube, magnetization reversal process, magnetic force microscopy

## Abstract

Magnetic nanowires, conceived as individual building blocks for spintronic devices, constitute a well-suited model to design and study magnetization reversal processes, or to tackle fundamental questions, such as the presence of topologically protected magnetization textures under particular conditions. Recently, a skyrmion-tube mediated magnetization reversal process was theoretically reported in diameter modulated cylindrical nanowires. In these nanowires, a vortex nucleates at the end of the segments with larger diameter and propagates, resulting in a first switching of the nanowire core magnetization at small fields. In this work, we show experimental evidence of the so-called Bloch skyrmion-tubes, using advanced Magnetic Force Microscopy modes to image the magnetization reversal process of FeCoCu diameter modulated nanowires. By monitoring the magnetic state of the nanowire during applied field sweeping, a detected drop of magnetic signal at a given critical field unveils the presence of a skyrmion-tube, due to mutually compensating stray field components. That evidences the presence of a skyrmion-tube as an intermediate stage during the magnetization reversal, whose presence is related to the geometrical dimensions of the cylindrical segments.

## 1. Introduction

Cylindrical magnetic nanowires (NWs) constitute a subject of thorough studies [1,2,3,4]. They have been envisioned as suitable elements to build spintronic devices [5,6] with limitless operation speed, as it has been put forward that it leads to the suppression of the Walker breakdown [7]. A variety of strategies have been implemented in order to achieve a controlled magnetization reversal with special focus on the domain wall pinning at designed locations, such as adding notches [8], non-magnetic spacers [9] or alternating changes in the diameter or length of the segments [10].

Lately, the cylindrical NWs are attracting increasing interest from the fundamental viewpoint [11] as their curved geometry can favor the emergence of topologically protected magnetization configurations, such as disruption of the continuity of the magnetization vector (Bloch point) [12,13]. This makes NWs appealing model systems to study the underlying physics of topologically protected magnetization textures. Recently, Fernandez-Roldán et al. theoretically described a topologically non-trivial configuration mediated magnetization reversal processes in magnetically soft nanowires with modulated diameter [14]. In this work, it is shown that magnetization reversal is initiated in segments with thicker diameters where vortices are predicted to nucleate and expand forming tubes, which subsequently transform into Bloch skyrmion-tubes (SkT). Other authors also predicted a SkT mediated magnetization reversal process in a nanowire of a Dzyaloshinskii-Moriya interaction (DMI) free material, where the nucleation of a hedgehog-antihedgehog pair governs the process [15].

Although magnetic skyrmions are considered to be two dimensional objects originated in systems with DMI [16,17], examples can be found in the literature where skyrmions are stabilized in the presence of perpendicular magnetocrystalline anisotropy (PMA) [18,19] or even, three dimensional skyrmionic configurations can be stabilized in confined systems in absence of both DMI and PMA [20]. On the other hand, experimental images combining two complementary techniques, X-ray holography and cryogenic Scanning Transmission X-ray Microscopy, have experimentally demonstrated the stabilization of SkT in FeGe lamellae [21].

However, the emergence of SkTs mediating the magnetization reversal process in DMI free nanostructures has not yet been experimentally observed. In this work, we present evidence of a SkT mediated reversal in nanowires with modulated diameter. Variable-Field Magnetic Force Microscopy (VF-MFM) images present an unusual loss of magnetic contrast which is attributed to the presence of a SkT for a narrow field range, when the nanowire is subjected to a sweeping external magnetic field.

## 2. Methods

Nanowire fabrication: FeCoCu NWs were fabricated by electrodeposition in aluminum oxide (AAO) membranes [1,22]. The membranes were obtained through pulsed hard anodization, keeping a constant voltage value (80 V) in a water based oxalic solution at 0 degrees for 10 min to create a protective oxide layer. Subsequently, the nanopores were aligned after the voltage has been slowly increased at 100 V and kept constant for 450 s. The modulations along the alumina pores were produced by alternating voltage pulses of 130 and 100 V for 5 and 150 s, respectively. Before growing the nanowires, the alumina layer at the bottom was removed by wet-chemical etching and a gold layer is sputtered as an electrode. Subsequently, the NWs are grown by DC electrodeposition using the following electrolyte: 0.12 M CoSO_4_, 0.05 M FeSO_4_, 0.01 M CuSO_4_, 0.16 M H_3_BO_3_, and 0.06 M C_6_H_8_O_6_. For the MFM measurements, the alumina was dissolved and the nanowires are washed and spread onto a silicon substrate by spin coating [8].

Magnetic Force Microscopy imaging: All the measurements were performed using a scanning force microscope from Nanotec Electronica S.L. (Madrid, Spain) controlled by the WSxM software [23] from WSxM solutions (Madrid, Spain) and Nanosensors PPP-MFMR magnetic probes from Nanoworld AG (Neuchâtel, Switzerland) as well as home-made cobalt sputtered probes in some cases. Amplitude modulation method was carried out enabling the phase-locked loop (PLL) to track the resonance frequency of the oscillating cantilever and the magnetic signal was therefore recorded in the frequency shift channel, in Hz.

Stray field simulation: The magnetic stray field distribution created by a single NW segment of 2 μm in length and 140 nm of diameter was simulated using COMSOL Multiphysics (Stocholm, Sweden). The nanowire was located in the middle of a simulated non-magnetic environment of 6 μm × 2.1 μm × 2.1 μm using discretized tetrahedra. The saturation magnetization of the NW was set to M_s_ = 1.4 × 10^15^ A/m and the additional parameters were extracted from the COMSOL library (setting the materials as iron for the NW and air for the environment). The stray field (magnetic flux density in normal direction to the plane that contains the nanowire, Bz) was quantified for different magnetic configurations, single domain (magnetization pointing to NW axis) and SkT with skyrmion radii 10, 20 and 30 nm.

## 3. Results and Discussion

### 3.1. Modulated Nanowires with Increasing Segment Length

**Figure 1:**
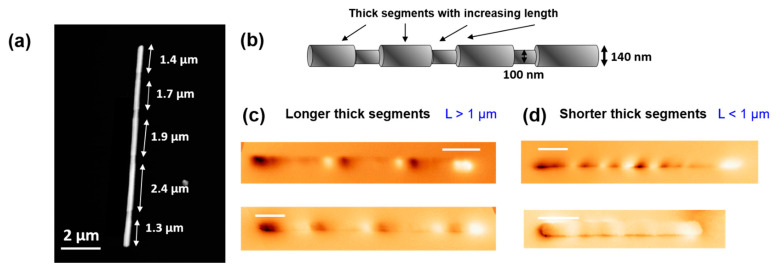
(**a**) Atomic Force Microscopy (AFM) topography image of a typical studied nanowire and (**b**) illustrative sketch of the diameter modulated structure showing the increasing segment length of the thick segments. Magnetic Force Microscopy images at remanence state of (**c**) nanowires with longer thick segments and (**d**) NW with thick segments shorter than 1 µm.

For the present study, nanowires of alternating diameter segments (d = 100 nm and D = 140 nm) have been fabricated as detailed in the methods section. Different trends can be found in these nanowires, as they are generally broken into smaller pieces during their release from the alumina membrane [1,24] and washing process, as shown in previous publications [9,10]. While the thicker segments possess an increasing length ranging between 200 nm to 2000 nm, the thinner ones are typically around 350 nm, see Figure 1a,b. The NW stoichiometry, namely Fe_28_Co_67_Cu_5_, gives rise to a very weak magnetocrystalline anisotropy, deriving from the *bcc* cubic symmetry, compared to the shape anisotropy resulting from their large aspect ratio.

Their overall magnetization is expected to be pointing in the axial direction, and hence, the main bright/dark contrast at the edges of the NW is due to a high concentration of magnetic charges (positive/negative) of this axial component. Moreover, the contrast along the nanowires reveals the existence of different relatively complex configurations. As MFM is mainly sensitive to the stray field, in most nanowires, additional bright/dark contrast is observed along the nanowire at the modulation sites. In addition, the modulation of the diameter induces the formation of flux-closure magnetization at the diameter transition sites, that gives rise to a bending effect of the axial magnetization. Remarkably, thick segments do present single or multivortex structure in some cases [10].

However, systematic MFM imaging revealed that two different trends can be found in such nanowires, seemingly related to the length of the thicker segments. We have noticed that while the reported flux-bending effect is in good agreement with the observed overall intermediate MFM contrast in many of the NWs (Figure 1c), in the shorter thick segments (L < 1 µm) this intermediate dim contrast is rather unexpected. Despite the complexity of the magnetization configuration, it is expected that longer thick segments possess a higher local shape anisotropy and generate a higher stray field at the diameter transition sites. This stray field is attenuated in the short thick segment nanowires, where the formation of vortices gives rise to a more complex MFM signal where the different components are added up (Figure 1d). In Supporting Information S1, we have added an illustrative MFM image, displaying a contrast compatible with the predicted formation of vortices in the middle of the segments.

### 3.2. Skyrmion-Tube Mediated Imaging of Magnetization Reversal Process Though Advanced MFM

**Figure 2:**
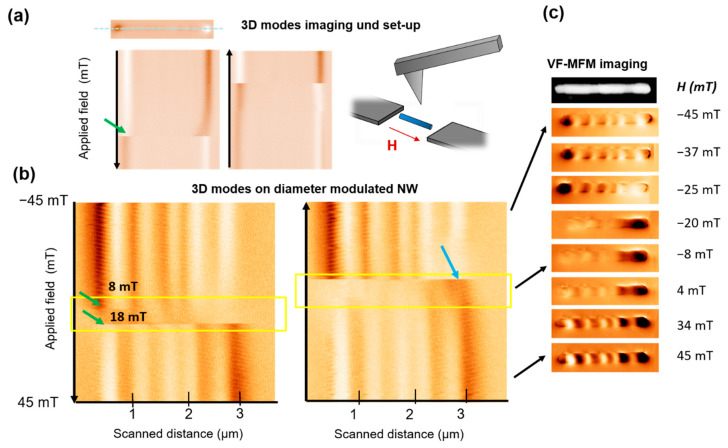
(**a**) Advanced MFM or 3D MFM operation modes on a single-domain magnetic nanowire. (**b**) 3D MFM image of a nanowire with modulated diameter. The area inside the yellow dashed square showing a dim signal is attributed to the presence of a skyrmion-tube. (**c**) MFM imaging sequence at changing field values, starting from positive applied field values.

Overall, MFM has proved to be a solid technique to study magnetization reversal process at the nanoscale [25,26,27]. In previous articles, the advanced VF-MFM modes (also called 3D modes) developed in our group [28] have proven to be a straightforward method to image the magnetization reversal processes in elongated nanostructures, study pinning events and reconstruct the hysteresis loop of individual elements [9,29,30]. For this operation mode, the nanowire is located and the same line is repeatedly scanned along the NW axis, while a magnetic field is applied in the same direction during each scan. This field is swept, ideally leading the nanowire from one saturated state to the opposite, while the changes in the signal associated to the magnetization of the nanowire are tracked in the resulting image. This is illustrated in Figure 2a for the simplest case, where a single domain nanowire (with constant diameter) reverses magnetization through a single giant Barkhausen jump (marked with a green arrow).

Coming back to the nanowires with short thick segment lengths (below 1 micron, such as the one shown in Figure 1d), 3D mode images shown in Figure 2b unveil an unusual behavior. While the initial and ending states display magnetic contrast near to the saturated state (not fully saturated as a much larger field is needed to align the magnetic moments in the diameter transitions along the axial direction), at intermediate stages an unexpected loss of magnetic signal is detected. The region has been marked in a yellow dashed square. The effect arises in a relatively large field range, starting at H = −20 mT, until the contrast is recovered at H = 4 mT and displaying reversed magnetization direction. Notice that the two branches are not entirely symmetrical. Additionally, VF-MFM images recorded at decreasing applied field (absolute) values, give some insight into the different stages that the nanowire is going through while the field is reversed from −45 to +45 mT (see Figure 2c). Notice that while the field is swept, the loss of contrast corresponding to the appearance of a SkT is less abrupt than the emergence of MFM signal. If one looks closely at the area enclosed in the yellow square, it becomes obvious that at the emergence of SkTs is taking place at slightly different field values for every segment. In fact, as it has been theoretically predicted, the magnetization configuration can evolve differently under applied field in different nanowire regions. This is well-illustrated by the region marked with a light blue arrow in Figure 2c, where the presence of a darker contrast indicates that SkT tube is not present in this segment. Conversely, the skyrmion-tube vanishes at a specific critical value, as we can see in both of the VF-MFM images displayed in Figure 2b.

Firstly, to interpret this loss of contrast the reader should bear in mind that MFM signal relates to the force gradient resulting from the tip and sample stray field interaction. To discard here possible imaging artifacts, such as variations between tip sample distance during the image acquisition (which would potentially result in a loss of contrast), the oscillation amplitude was recorded and no changes were detected. Notice that in the Amplitude-Modulation mode (AM-AFM), the amplitude is used as a feedback parameter to obtain a topographic image, and therefore, a uniform oscillation amplitude value ensures no changes in tip-sample distance (see Supporting Information S2 for more details). Moreover, the change in the contrast is not simultaneous along a particular scan line, on the contrary, there are some features along the same line what also discard the sudden change of the tip properties and reinforce the idea of independent processes occurring in different segments.

Another important fact, is that while the contrast of planar samples usually has a straightforward interpretation, in three dimensional nanostructures (such as the studied cylindrical nanowires) different contributions are added together in the same image. All this evidence supports the idea of a SkT nucleation that mediates the magnetization reversal process, as theoretically predicted [14], where the stray field components coming from the NW core and shell parts, compensate and eventually cancel one another.

**Figure 3:**
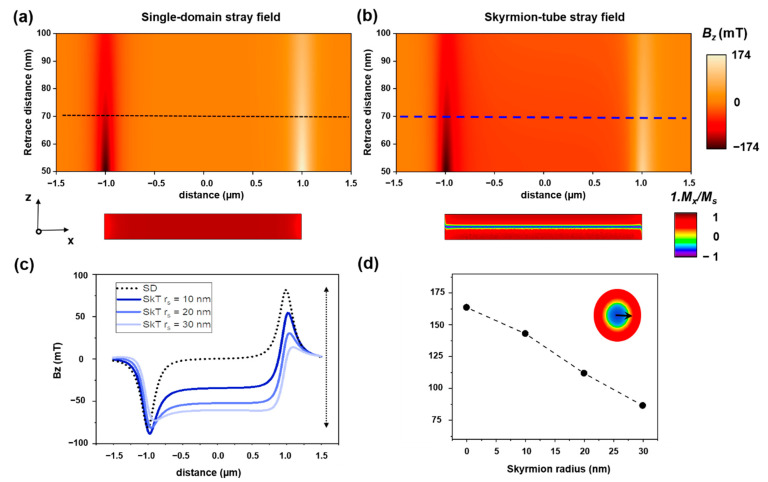
Simulated stray field map between 50 and 100 nm from the nanowire for a (**a**) single domain and (**b**) a skyrmion-tube configuration in a magnetic nanowire, for a skyrmion radius r_s_ = 20 nm. (**c**) Corresponding stray field contrast profiles at 70 nm of the nanowire surface. (**d**) Dependency of the stray field “contrast” on the skyrmion radius, defined in the inset. The line is drawn as a guide to the eyes.

To qualitatively illustrate this idea, the corresponding stray field generated by a single domain and a SkT magnetization configurations were simulated and compared (Figure 3a,b) using COMSOL Multiphysics, Stocholm [31]. For simplicity, a 2 µm long NW of 140 nm of diameter was simulated, without considering any diameter modulations. The calculated stray field maps give an idea of the stray field generated by the two configurations at a distance range comparable to the *retrace* distance for the MFM data acquisition. The profiles in Figure 3c allow direct comparison between the stray field values obtained at a typical distance of 70 nm from the nanowire surface. The data showcases how the stray field contrast decreases due to the contribution of the opposite stray field generated by the opposite core magnetization.

For the displayed images in Figure 3a,b, a skyrmion with radius of r_s_ = 20 nm was considered. For larger skyrmion radius, the effect of the stray field reduction is even more pronounced. To illustrate this, we have plotted the “stray field variation” (Bz (maximum)-Bz(minimum)) and plotted it versus skyrmion tubes with different skyrmion radii (Figure 3d). Notice that the case r_s_ = 0 corresponds with a single domain configuration.

### 3.3. Differences in the Magnetization Reversal Process

One of the main assets of VF-MFM is its capability to provide qualitative and partial quantitative information of the magnetization reversal process of individual nanowires in a relatively fast manner, as compared to other imaging techniques. Because each particular nanowire could present small crystallographic or geometrical differences as a result of small structural defect formation or breaking during preparation, this method constitutes a suitable tool to perform broader studies and identify variations in the magnetization reversal processes within nanowires fabricated in a single batch.

**Figure 4:**
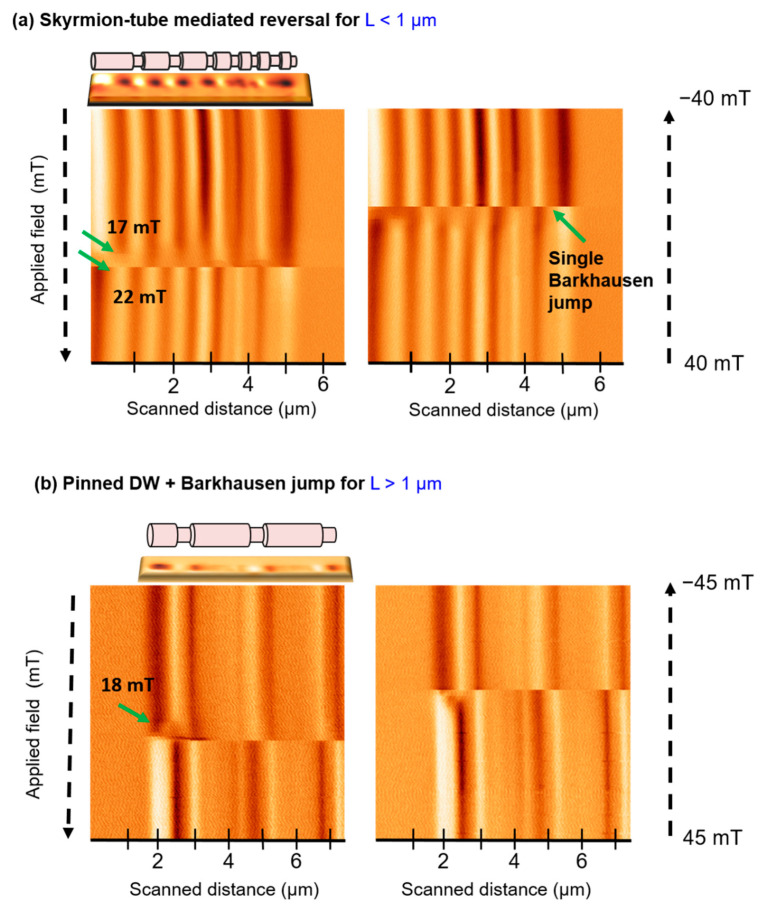
Advanced (3D-mode) MFM image of different magnetization reversal processes. (**a**) Skyrmion-tube mediated reversal. (**b**) Domain wall pinning followed by a Barkhausen jump.

Skyrmion-tube mediated magnetization reversals have been detected in several nanowires (see Figure 4a). Here, we observe an MFM signal loss before the magnetization is fully reversed (near the demagnetized state), as in Figure 2b, although in this case, the skyrmion tube configuration persists in a smaller range of fields. Though in general terms, a repeated hysteresis loop in same NW leads to the same type of magnetization reversal process, small quantitative variations can be observed depending on the specific measurement conditions, such as the scanning height, the scan speed or the previous magnetic history. On the other hand, the micromagnetic simulations [14] predict a very fast formation and propagation of a skyrmion-tube, unlike the pinning effect we can observe in our measurement, were the configuration is stable despite the relatively slow scanning speed (around 100 µm/s) in comparison to the fast propagation of DW (in the order of 100 m/s) and for several applied field values. A similar effect studied in reference [30] was attributed to the presence of local defects in nanowires that slow down the magnetization reversal. Additionally, a closer look at the SkT area reveals that the contrast in the images is compatible with the formation of such configuration exclusively in thicker wire segments, as predicted in reference [14].

In addition to skyrmion-tubes, in the same NW, the shorter segments can also reverse magnetization through a single Barkhausen jump (Supporting Information S3), where the axial magnetization reverses direction in a single event, when a domain wall propagates from one NW end to the opposite. Finally, similarly to other nanowires with variations in the diameter [30], domain wall pinning can be also detected in this type of NWs (see Figure 4b). Here, only the left end of a very long nanowire has been scanned due to the difficulties to scan bigger sizes through this operation mode. However, it enables us to observe the nucleation and pinning of a domain wall at the first diameter variation site, prior to its depinning and subsequent propagation. In the case of longer thick segment nanowires (L > 1 µm), a single Barkhausen jump constitutes the most common magnetization reversal mechanism (Figure 4b).

From the studied data, we hypothesize that the formation of a skyrmion-tube and its stabilization is more favorable in nanowires with shorter segments, as the local shape anisotropy is smaller and the creation closure configurations is energetically less costly.

## 4. Conclusions

The magnetization reversal processes of FeCoCu nanowires with diameter modulations has been investigated using VF-MFM advanced imaging modes. These modes enable us to observe differences in the remanent magnetization configurations as well as in the low-field magnetization reversal processes, depending on the thicker segment length. In NWs with longer thick segments, the obtained imaging data are compatible with a skyrmion-tube mediated magnetization reversal process, as predicted by micromagnetic simulations in nanowires of comparable dimensions. To the best of our knowledge, the MFM data presented constitute the first experimental evidence of a skyrmion-tube mediated magnetization reversal process in cylindrical nanowires.

## Data Availability

The data presented in this study are available upon reasonable request to the corresponding author.

## Supplementary Materials

The following are available online at https://www.mdpi.com/article/10.3390/ma14195671/s1, Figure S1: (a) Simulated magnetization configuration of a diameter modulated nanowire, showing vortex formation at intermediate positions. Reprinted with permission from Rodríguez, L. A. et al. Quantitative Nanoscale Magnetic Study of Isolated Diameter-Modulated FeCoCu Nanowires. ACS Nano 10, 9669–9678 (2016), Copyright 2016 American Chemical Society. (b) MFM image of NW with longer thick segments, displaying intermediate contrast related to vortex formation, marked with arrows, Figure S2: (a) 3D mode image of the magnetic signal showing evidence of skyrmion tube. (b) Corresponding oscillation amplitude data. (c) Oscillation amplitude profiles taken from the image in (b). Figure S3: 3D mode imaging of the magnetization reversal happening through a single Barkhausen jump.
