# Supplementary material for: Evidence of Skyrmion-Tube Mediated Magnetization Reversal in Modulated Nanowires
Source: Materials (Basel). 2021 Sep 29;14(19):5671. doi: 10.3390/ma14195671 (PMC8509997; doi:10.3390/ma14195671)
Supplement: Supplementary file 1 [file materials-14-05671-s001.zip › materials-1382608-supplementary.pdf]

Supporting Information

S1 Evidence of vortex formation at intermediate positions:

The formation of vortices at intermediate positions in diameter modulated nanowires was predicted by simulations in ref [10]. The MFM image in Figure S1 experimentally demonstrates the presence of vortices at these intermediate positions.

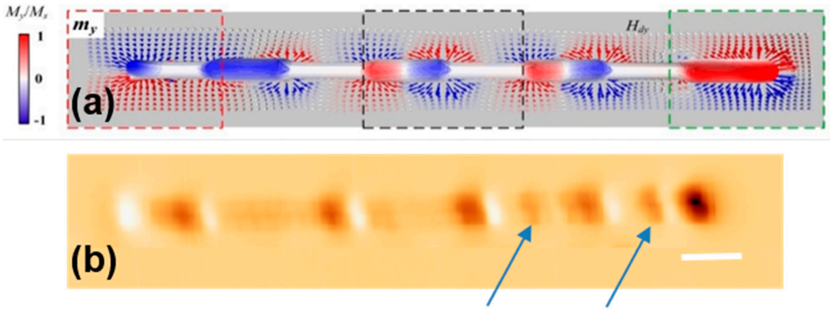

Figure S1. (a) Simulated magnetization configuration of a diameter modulated nanowire, showing vortex formation at intermediate positions. Reprinted with permission from Rodríguez, L. A. *et al.* Quantitative Nanoscale Magnetic Study of Isolated Diameter-Modulated FeCoCu Nanowires. *ACS Nano* **10**, 9669–9678 (2016), Copyright 2016 American Chemical Society. **(b)** MFM image of NW with longer thick segments, displaying intermediate contrast related to vortex formation, marked with arrows.

S2 Negligible variations in the oscillation amplitude signal during 3D imaging:

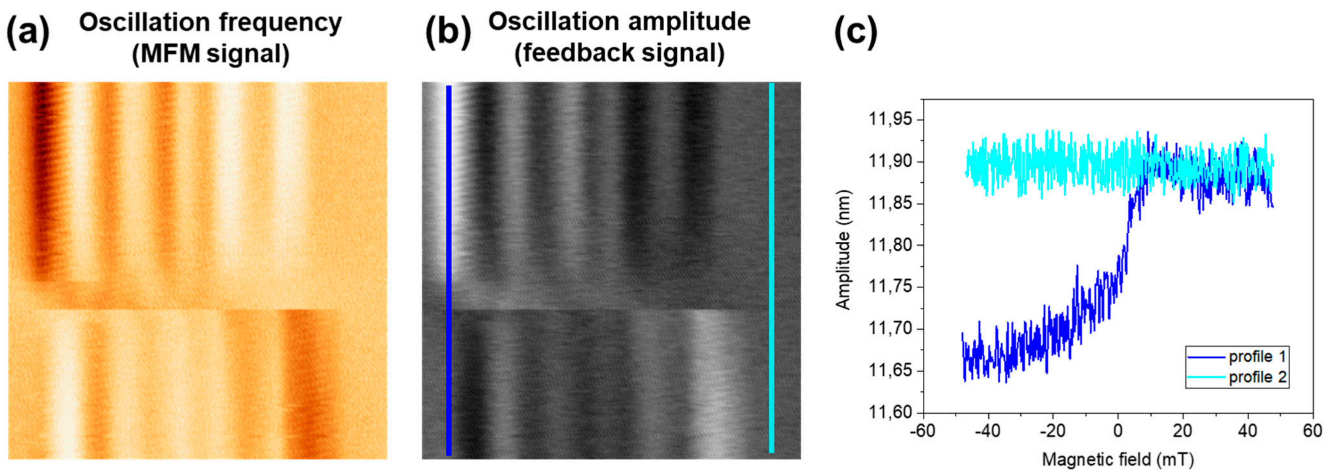

Figure S2. (a) 3D mode image of the magnetic signal showing evidence of skyrmion tube. **(b)** Corresponding oscillation amplitude data. **(c)** Oscillation amplitude profiles taken from the image in (b).

S3 Single Barkhausen jump in short thick segment nanowires:

Although many nanowires with short thick segments present skyrmion-tube mediated magnetization reversal processes, in some cases we have also detected a single-barkhausen jump mediated magnetization reversal, as shown in the 3D mode images displayed in Figure S3.

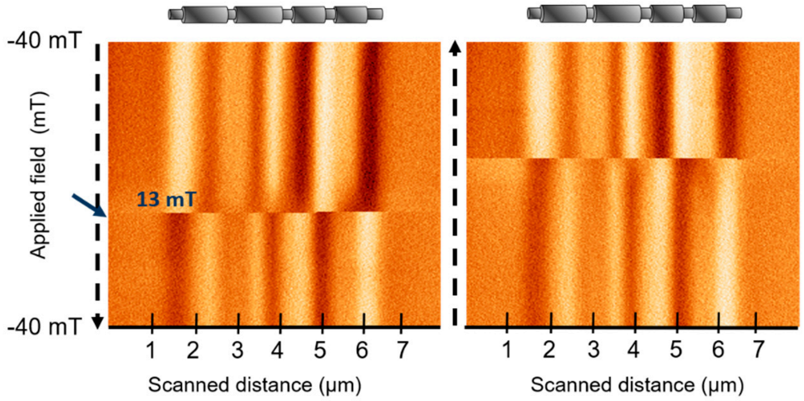

Figure S3. 3D mode imaging of the magnetization reversal happening through a single Barkhausen jump.
